# AI-Enhanced Electrochemical Sensing Systems: A Paradigm Shift for Intelligent Food Safety Monitoring

**DOI:** 10.3390/bios15090565

**Published:** 2025-08-28

**Authors:** Yuliang Zhao, Tingting Sun, Huawei Zhang, Wenjing Li, Chao Lian, Yongqiang Jiang, Mingyue Qu, Zhongpeng Zhao, Yuhang Wang, Yang Sun, Huiqi Duan, Yuhao Ren, Peng Liu, Xulong Lang, Shaolong Chen

**Affiliations:** 1 School of Control Engineering, Northeastern University at Qinhuangdao, Qinhuangdao 066000, China; zhaoyuliang@neuq.edu.cn (Y.Z.); 2302121@stu.neu.edu.cn (T.S.);; 2 State Key Laboratory of Pathogen and Biosecurity, Academy of Military Medical Sciences, Beijing 100071, China; 3 The PLA Rocket Force Characteristic Medical Center, Beijing 100088, China; 4 State Key Laboratory of Pathogen and Biosecurity, Key Laboratory of Jilin Province for Zoonosis Prevention and Control, Changchun Veterinary Research Institute, Chinese Academy of Agricultural Sciences, Changchun 130122, China

**Keywords:** electrochemical biosensors, artificial intelligence, pathogen detection, food safety

## Abstract

Artificial intelligence (AI) is transforming electrochemical biosensing systems, offering novel solutions for foodborne pathogen detection. This review examines the integration of AI technologies, particularly machine learning and deep learning algorithms, in enhancing sensor design, material optimization, and signal processing for detecting key pathogens such as *Escherichia coli*, *Salmonella*, and *Staphylococcus aureus*. Key advancements include improved sensitivity, multiplexed detection, and adaptability to complex environments. The application of AI to the design of recognition molecules (e.g., enzymes, antibodies, aptamers), as well as to electrochemical parameter tuning and multicomponent signal analysis, is systematically reviewed. Additionally, the convergence of AI with the Internet of Things (IoT) is discussed as a pathway to portable, real-time detection platforms. The review highlights the pivotal role of AI across multiple layers of biosensor development, emphasizing the opportunities and challenges that arise from interdisciplinary integration and the practical deployment of IoT-enabled technologies in electrochemical sensing systems. Despite significant progress, challenges remain in data quality, model generalization, and interpretability. The review concludes by outlining future research directions for building robust, intelligent biosensing systems capable of supporting scalable food safety monitoring.

## 1. Introduction

Food safety, particularly the prevention of infectious diseases caused by foodborne pathogens such as *Salmonella*, *Escherichia coli*, and *Listeria monocytogenes*, has become a persistent global challenge, impacting both public health and the stability of food supply chains. According to estimates by the World Health Organization (WHO), approximately 600 million people worldwide suffer from foodborne illnesses each year, resulting in an estimated 420,000 deaths [1]. A significant proportion of these cases occur in low- and middle-income countries (LMICs), thereby exacerbating public health burdens and intensifying demands for detection technologies that are not only accurate and timely but also accessible and field-deployable. Conventional detection methods, including microbial culture, polymerase chain reaction (PCR), and enzyme-linked immunosorbent assay (ELISA), although exhibiting good sensitivity and specificity in detecting foodborne pathogens such as *Salmonella* spp. [2], *Escherichia coli* [3], and *Staphylococcus aureus* [4], are hindered by complex protocols, prolonged detection times, and a high dependence on sophisticated equipment and trained personnel [5]. These limitations make them insufficient to meet the growing demand for rapid, on-site, and high-throughput pathogen detection across modern food production systems. Particularly in scenarios involving complex sample matrices or resource-constrained settings, achieving precise identification and real-time monitoring of diverse pathogens remains a critical bottleneck in the development of sensing technologies.

Against this backdrop, electrochemical biosensors have demonstrated considerable promise in foodborne pathogen detection [6]. However, their performance in real-world environments is frequently compromised by inadequate stability, selectivity, and reproducibility, which severely restricts both output efficiency and the scope of practical deployment [7]. A closer examination reveals that sensor performance is not solely determined by individual components but rather by the synergistic interaction of multiple functional layers—specifically, the structural modulation of electrode materials, the specificity conferred by biorecognition elements, and the accuracy of signal transduction mechanisms [8]. As such, a comprehensive optimization strategy that simultaneously enhances the material, recognition, and signal layers has become central to ongoing research efforts.

The structural design of sensor materials forms the foundational basis for electrode functionality, directly influencing conductivity, operational stability, and interfacial reaction efficiency. The microstructure, compositional configuration, and surface modification of electrode materials profoundly impact electrochemical reaction kinetics and reversibility, as well as the sensor’s sensitivity and resilience in complex sample environments. Particularly when electrodes are fabricated through manual techniques, inconsistencies in preparation often result in significant batch-to-batch variation, undermining signal stability and experimental reproducibility [9]. In addition, the thermodynamic stability at the electrode–electrolyte or electrode–sample interface and the role of nanostructured elements in facilitating target recognition and signal amplification are pivotal factors influencing overall sensing performance. While enhancing the physicochemical properties of materials can partially mitigate performance fluctuations, sustained improvement requires deeper control over interfacial reaction mechanisms and the coordinated design of multifunctional materials [10,11].

Biorecognition elements constitute the core of electrochemical biosensors, with their binding specificity directly determining the accuracy and sensitivity of pathogen detection. Conventional recognition molecules, including antibodies, aptamers, nucleic acid probes, and enzymes, each possess distinct advantages and inherent limitations, while recent studies have increasingly incorporated nanomaterials to enhance interfacial signal transduction efficiency. Beyond mere performance characterization, the optimization paradigm is shifting from empirically driven approaches toward AI-assisted molecular screening and structural design, wherein machine learning models predict binding sites, affinities, and environmental stability to enable the more efficient development of recognition elements. This strategy not only shortens the research and development cycle but also enhances detection reliability under complex sample conditions.

Traditional electrochemical detection methods have long played a pivotal role in foodborne pathogen identification, such as *Escherichia coli*, *Salmonella* spp., and *Listeria monocytogenes* [12,13,14]. However, they fundamentally rely on manually defined parameters and empirical combinations of materials and recognition elements, making them susceptible to external interference and sample variability. When confronted with issues such as complex sample matrices, background noise, and weak target signals, these methods often lack the adaptability required to maintain reliable performance, resulting in unstable responses, poor reproducibility, and insufficient specificity [15]. Moreover, the preparation of electrode modification materials remains largely empirical, with limited access to data-driven, quantitative optimization strategies [16]. This reliance on experience-based workflows results in inefficient experimental design and poor reproducibility, posing significant barriers to the large-scale deployment and sustained operation of electrochemical sensors in real-world applications.

In this review, “environmental factors” specifically refer to external conditions and system status that affect the performance of electrochemical sensors, including physical factors (temperature, humidity, pressure), chemical factors (sample pH, ionic strength, matrix components such as organic substances and additives in food), and sensor status (e.g., contamination, oxidation, stability of biomolecule immobilization on the electrode surface, and electrolyte aging). This list is not exhaustive, and in practical applications, further confirmation is required to exclude relevant interferences as much as possible. Notably, artificial intelligence techniques hold significant potential in mitigating the impacts of these environmental factors, enabling the acquisition of relatively standardized and reliable data through compensation, correction, or adaptive adjustment strategies.

With continued technological evolution, artificial intelligence (AI), particularly machine learning (ML), deep learning (DL), optimization algorithms, and graph neural networks (GNNs), offers transformative solutions to the aforementioned challenges involving multi-objective and multi-parameter coordination. At the molecular level, AI can facilitate sequence optimization, structural prediction, and the functional screening of enzymes, antibodies, and aptamers [17]. At the materials level, it enables the global modulation of electrode configurations, conductivity, and immobilization strategies [18]. Furthermore, by modeling nonlinear features in electrochemical signals, AI enables anomaly detection, background correction, and multiplexed target recognition [19]. This data–structure–function integration paradigm, driven by AI, is reshaping the foundational logic of electrochemical sensor design.

**Figure 1:**
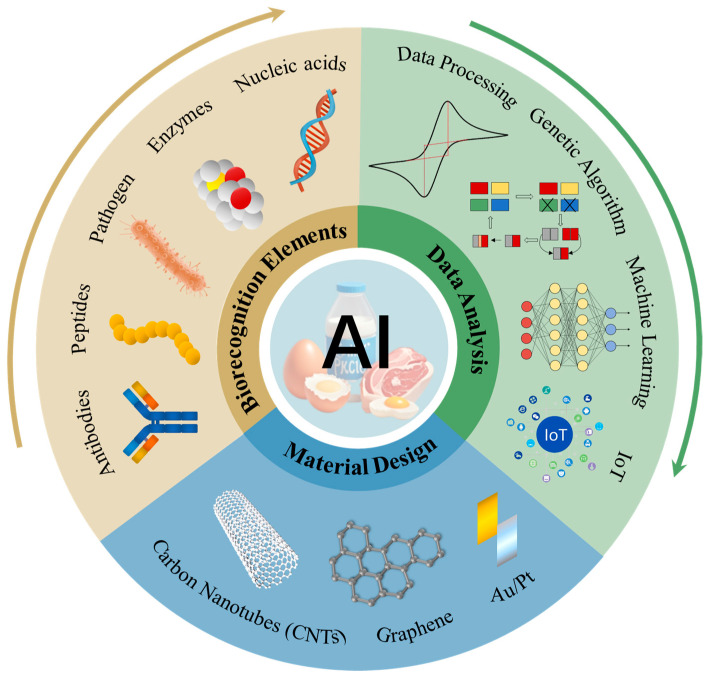
Integration of artificial intelligence (AI)-driven electrochemical biosensors into foodborne pathogen detection systems.

Concurrently, the integration of AI with the Internet of Things (IoT) is accelerating the advancement of intelligent sensor deployment and remote interaction capabilities. Through wireless communication and edge AI models, sensors now support high-frequency, low-power data acquisition and real-time analysis, while also enabling environmental awareness, adaptive control, and autonomous decision-making [20]. This forms the technological underpinning for “unattended monitoring–intelligent alerting–systemic feedback” workflows, which are particularly well-suited to multi-node sensing scenarios throughout the entire food supply chain, including agriculture, transportation, and processing stages, as shown in Figure 1.

Most existing reviews predominantly emphasize a single technological dimension or focus on detection strategies targeting individual pathogens [21,22]. For example, some exclusively address the application of AI in optimizing biorecognition elements [23], while others concentrate on material advancements in electrochemical biosensors for food analysis [24], yet they fall short of offering a systematic synthesis of multi-level synergistic optimization spanning molecules, materials, signal transduction, and system deployment. Furthermore, many studies have not thoroughly explored the convergence of the Internet of Things (IoT) with AI, nor have they adequately examined issues of deployability within the complex landscape of food supply chains. In contrast, the present review broadens both scope and depth: adopting a full-chain perspective, we systematically summarize the mechanisms and optimization strategies through which AI enhances electrochemical biosensors for foodborne pathogen detection, encompassing four critical dimensions—biorecognition molecule design, sensor material engineering, signal processing algorithms, and field-deployable models. By integrating representative frameworks with real-world case studies, we further propose an intelligent, IoT-enabled, multi-node monitoring architecture, thereby laying a novel theoretical foundation and practical pathway toward the construction of high-performance, scalable food safety detection systems.

This review focuses on the AI-driven evolution and systemic transformation of electrochemical biosensors in the context of foodborne pathogen detection. We provide a comprehensive analysis of the multidimensional roles of AI in the design of biorecognition molecules, the optimization of sensing materials, the processing of electrochemical signals, and field-level deployment. Representative models, algorithms, and practical case studies are summarized to illustrate performance advantages and application scenarios. Additionally, we assess the key challenges currently facing AI-enabled sensing systems, such as data quality, model interpretability, multi-objective coordination, and operational stability in real-world environments, and explore future directions for research and development. Through this analysis, we aim to offer both theoretical insight and practical pathways for constructing high-performance, scalable, and intelligent food safety detection systems.

## 2. Adaptability of Artificial Intelligence (AI) Methodologies to Electrochemical Sensing

AI, as a collective suite of technologies dedicated to complex data modeling and task optimization, has witnessed rapid integration into disciplines such as materials science, bioengineering, and sensor technology. The multilayered architecture, intricate signal profiles, and pronounced environmental sensitivity of electrochemical biosensing systems present formidable challenges in terms of design, regulation, and signal interpretation, involving multi-objective, multivariate, and highly nonlinear variables. Traditional experiment-driven methodologies often fall short in addressing such complexity, whereas the incorporation of AI offers transformative solutions for task modeling, performance optimization, and dynamic system responsiveness.

### 2.1. Mainstream AI Methodologies

AI methods, as the foundational tools for data-driven modeling and system optimization, are typically categorized according to their learning paradigms and task objectives. Within electrochemical biosensing systems, diverse AI techniques can be strategically deployed across sub-tasks such as biorecognition element design, material parameter optimization, signal modeling, and system control.

#### 2.1.1. Supervised Learning

**Figure 2:**
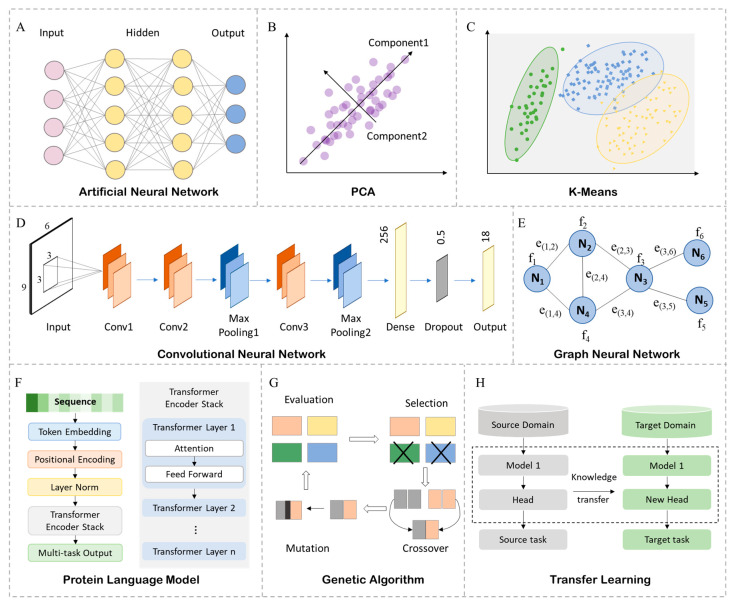
Algorithms commonly employed in the design of electrochemical biosensing systems. (**A**) Artificial Neural Network. (**B**) Principal Component Analysis. (**C**) K-Means Clustering. (**D**) Convolutional Neural Network. (**E**) Graph Neural Network. (**F**) Protein Language Model. (**G**) Genetic Algorithm. (**H**) Transfer Learning.

Supervised learning remains the most widely utilized AI paradigm, relying on large volumes of labeled data for model training. These methods center on mapping relationships between input features and output responses, making them particularly suitable for classification and regression tasks. In electrochemical sensing, they are commonly employed for target concentration prediction, signal pattern recognition, and pathogen classification. Representative algorithms include Support Vector Machines (SVMs), Random Forests (RFs), and Artificial Neural Networks (ANNs) [25,26,27]. ANNs, in particular, emulate the synaptic connections of biological neurons through multilayered networks comprising input, hidden, and output layers, enabling powerful nonlinear feature extraction capabilities (Figure 2A). While supervised learning models offer clear architectures and high computational efficiency, their performance can degrade in the presence of insufficient labeling or uneven data distributions.

#### 2.1.2. Unsupervised Learning

Unsupervised learning dispenses with the need for labeled data, excelling in uncovering latent structures and intrinsic patterns within datasets. It is predominantly applied to feature dimensionality reduction, cluster analysis, and anomaly detection, which is especially valuable in tasks such as the compression of high-dimensional electrochemical signals, baseline drift recognition, and background noise elimination. Commonly employed algorithms include Principal Component Analysis (PCA) (Figure 2B), t-distributed Stochastic Neighbor Embedding (t-SNE), and clustering approaches such as K-means and Density-Based Spatial Clustering of Applications with Noise (DBSCAN) (Figure 2C) [28,29,30]. While particularly practical in unlabeled scenarios, the outputs of these models typically lack interpretable labels and thus often require integration with subsequent supervised frameworks.

#### 2.1.3. Deep Learning

DL leverages multilayered neural networks to construct hierarchical, nonlinear representations of complex features. Electrochemical signals, often unstructured in nature, such as current–time curves, voltage–current response plots (e.g., cyclic voltammetry (CV), differential pulse voltammetry (DPV)), and electrochemical impedance spectra (EIS), are typical examples. In addition, relevant studies also involve other types of data, such as electrode surface micrographs (e.g., Scanning Electron Microscope (SEM) images, a type of surface characterization technique) and nucleic acid/protein sequences (biomolecular sequences that determine biorecognition specificity, regulate target binding affinity, and mediate signal transduction), all of which present dynamic and structurally diverse characteristics. Convolutional Neural Networks (CNNs) are widely adopted in image-based material recognition and electrochemical signal interpretation (Figure 2D) [31,32], while Transformer-based sequence models are increasingly applied in the temporal modeling of electrochemical data and the functional prediction of biomolecular sequences [33,34]. Although DL models possess formidable feature extraction capabilities and circumvent the limitations of manual feature engineering, they are computationally intensive and demand substantial training datasets of high quality.

#### 2.1.4. Generative and Optimization Algorithms

These methodologies focus not on direct prediction but on solution space exploration and multi-objective function optimization. They are particularly useful in molecular structure design, combinatorial material screening, and sensor parameter tuning. Representative algorithms include Genetic Algorithms (GAs), Bayesian Optimization, and Reinforcement Learning (RL) [35,36,37,38]. GA, inspired by natural selection and mutation mechanisms, is suited for large-scale combinatorial searches in protein sequence design and nanostructure engineering (Figure 2G). Bayesian Optimization excels in efficiently locating global optima under costly evaluation conditions, facilitating the rapid optimization of electrode materials or process parameters. RL enables the development of adaptive detection strategies that evolve based on environmental feedback. Compared to predictive models, these algorithms are inherently more decision-oriented and constitute critical tools for resolving trade-offs in multi-objective optimization problems.

#### 2.1.5. Language Models and Graph Neural Networks

Recent advances in protein language models (e.g., the Evolutionary Scale Modeling (ESM) series) and GNNs have demonstrated groundbreaking capabilities in biomolecular modeling, particularly for optimizing recognition elements. Language models treat protein or nucleic acid sequences as “biological language,” employing self-supervised learning to capture high-order statistical dependencies between amino acids or nucleotides. As visualized in Figure 2F, these Transformer-based models first map sequences to embedded representations augmented with positional information, normalized by an initial layer norm to unify input distributions. Stacked Transformer encoder layers then process these features—each layer combines multi-head attention (for long-range residue interactions) and feed-forward networks, with additional layer norms per sub-module to enable stable deep training [39]. GNNs, on the other hand, are designed to process graph-structured data such as molecular topologies or crystal structures of electrode materials (Figure 2E) and are highly effective in conformation modeling and the prediction of physical properties [40]. Despite their structural complexity and computational demands, these models offer substantial advantages in molecular optimization and pathway elucidation.

#### 2.1.6. Few-Shot and Transfer Learning

In practical electrochemical sensing applications, limited experimental resources and data scarcity in specific scenarios often hinder the training of deep models. Few-Shot Learning and Transfer Learning have emerged as effective strategies to overcome such limitations. Few-Shot Learning enables the extraction of target distributions from minimal training samples through optimized network architectures, while Transfer Learning facilitates task adaptation by leveraging pretrained models (Figure 2H) [41,42]. These approaches exhibit considerable promise in signal calibration, environmental disturbance compensation, and cross-platform model migration, making them indispensable tools for building deployable and generalizable AI models.

In summary, the methodological landscape of AI is evolving from data modeling to structural generation and from static prediction to dynamic optimization. Each algorithmic class offers unique strengths, and its effective deployment demands task-specific customization based on data characteristics and system constraints. Together, these techniques provide a robust methodological and strategic foundation for advancing electrochemical biosensing systems.

### 2.2. Task-Specific Adaptation Analysis in Electrochemical Sensing

As a complex system characterized by the coordinated operation of multiple modules and objectives, electrochemical biosensing platforms rely on meticulous design and dynamic regulation across various hierarchical layers—from biorecognition units and material interfaces to signal acquisition and system-level integration. The heterogeneity of sub-tasks in terms of data structures, modeling requirements, and optimization goals necessitates a tailored application of AI methodologies with distinct functional capabilities [43].

Table 1 provides a comparative summary of mainstream AI methods and their corresponding suitability to different modules within electrochemical sensing systems. It underscores the task-specific deployment strategy of AI in this domain, reflecting a “task-driven fusion” paradigm.

In practical implementation, these AI methodologies are rarely deployed in isolation. Instead, they are frequently integrated through hybrid model architectures to collaboratively address the diverse tasks within the system [44]. For instance, aptamer screening can be initiated using language models for preliminary selection, followed by the optimization of thermal stability and binding specificity via GA [45]. Similarly, in modeling electrochemical signals and image-based data, combinations of CNN and RF are often employed to balance deep feature extraction with interpretability in decision-making [46,47].

Moreover, the deployment of AI methodologies must be context-aware, aligning model selection with the specific application scenarios of electrochemical sensing systems, while balancing model performance against practical constraints in computational resources. Several key dimensions guide method selection, as follows:

- Data Volume and Quality: In scenarios characterized by limited datasets or sparse labeling, strategies such as Few-Shot Learning, Transfer Learning, or lightweight model architectures are preferable to avoid overfitting and non-convergent training;
- Model Accuracy and Interpretability: In safety-critical domains such as foodborne pathogen detection, models must not only achieve high predictive accuracy but also provide transparent, traceable decision pathways to ensure regulatory compliance and operational trust;
- Computational Constraints and Deployment Platforms: For edge computing or field-deployable systems, models should exhibit fast inference speeds and low memory usage. Techniques such as model compression and knowledge distillation can be employed to reduce deployment barriers;
- Task Complexity and Environmental Dynamics: For systems dealing with multi-objective optimization or dynamically evolving environments, RL and adaptive control algorithms enhance robustness and generalizability across varying conditions;
- Model Update and Iteration Mechanisms: Long-term operational systems must support online model updates, incremental learning, and failure prediction to ensure sustained performance and reliability.

Thus, the effective application of AI in electrochemical sensing must transcend the paradigm of generic technology transfer and instead adhere to a task-oriented, data-aware, and resource-aligned algorithmic logic. Only by clearly delineating the adaptation boundaries and synergistic mechanisms of each method can the core advantages of AI—enhanced sensitivity, intelligence, and system robustness—be fully actualized in next-generation biosensing platforms.

## 3. Construction of High-Performance Electrochemical Biosensing Systems

In the pursuit of high-performance electrochemical biosensing systems, enhancing individual components in isolation often proves insufficient to address the multifaceted challenges encountered in real-world applications. The true determinants of system efficacy and adaptability lie in the synergistic optimization across multiple dimensions—including biorecognition elements, material architectures, parameter configurations, and signal interpretation strategies. Consequently, the integration of AI transcends the role of incremental performance enhancement, permeating every stage from molecular design to system-level integration. This holistic approach fosters the co-evolution of sensor capabilities across hierarchical layers. This section systematically delineates this optimization trajectory, beginning with AI-assisted biorecognition engineering, followed by algorithm-guided material and parameter tuning, intelligent signal analysis, and culminating in the development of integrated AI-IoT platforms, thereby elucidating how AI catalyzes comprehensive performance transformation from molecular foundations to system architecture.

### 3.1. Intelligent Optimization of Biorecognition Molecules

In electrochemical biosensing, the efficacy of biorecognition elements fundamentally governs the upper limits of sensitivity, selectivity, and stability. From natural enzyme systems to recombinant antibodies and in vitro-selected nucleic acid aptamers, these molecules differ structurally and mechanistically, yet their functional performance is universally dictated by intricate couplings among sequence information, three-dimensional conformation, and environmental interactions [48]. Conventional molecular engineering—reliant on empirical mutagenesis, directed evolution, and high-throughput screening—is often constrained by limited efficiency and prohibitive cost, rendering it ill-suited for the rapid development of novel targets [49]. The advent of AI is progressively dismantling these limitations, enabling end-to-end molecular design spanning from sequence generation to functional prediction and environmental adaptation.

**Figure 3:**
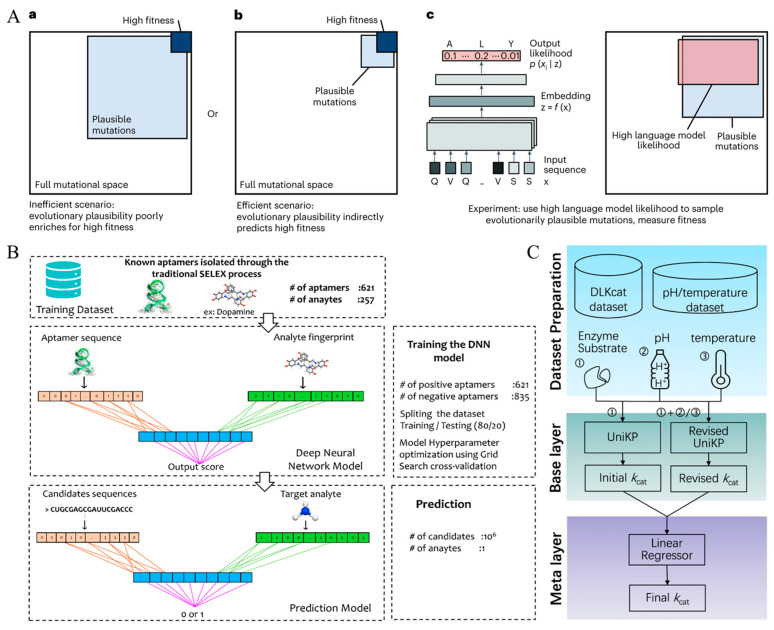
Optimization Process of Biomolecules Using AI Technology. (**A**) Protein language models guiding evolutionary optimization. (**a**) Rare high-fitness mutations within the evolutionarily plausible space. (**b**) High-fitness mutations more common among plausible mutations. (**c**) Protein language models capturing evolutionarily plausible amino acid patterns. Reproduced with permission from reference [54]. (**B**) A deep learning-SELEX model incorporating a Convolutional Neural Network (CNN) module for feature extraction from aptamer sequences. Reproduced with permission from reference [60]. (**C**) The EF-UniKP two-layer framework predicts enzymatic catalytic efficiency (*k*_cat_) based on pH and temperature. Reproduced with permission from reference [65].

At the core of any functional phenotype lies the three-dimensional structure encoded by the molecule’s primary sequence. While traditional structure determination techniques such as X-ray crystallography and nuclear magnetic resonance offer high resolution, their time-intensive and sample-demanding nature hampers scalability [50,51]. The emergence of AI—particularly DL and natural language processing—has made structure inference from sequence both feasible and generalizable, facilitating rational mutagenesis and predictive modeling. A seminal development in this domain is the rise of protein language models [52,53], which treat amino acid sequences as semantic strings and learn statistical dependencies among residues through self-supervised training. The ESM series, notably ESM-1b and ESM-1v, exemplifies this approach, uncovering latent “linguistic rules” governing structural stability and binding affinity. In a representative study, Hie et al. employed ESM to score single-point mutations, constructed a combinatorial mutant library, and achieved a 160-fold increase in binding affinity along with enhanced thermostability and neutralization efficacy, without any reliance on antigen structural information (Figure 3A) [54], thus showcasing its applicability in rapid, target-agnostic antibody design.

To integrate structural constraints into sequence modeling, ESM-IF1 incorporates AlphaFold-predicted structures, enabling structure-conditioned sequence generation. Shanker et al. demonstrated the efficacy of this model in engineering antibody mutants that retained high affinity and conformational stability against diverse viral variants, underscoring its capacity for cross-target generalization [55]. Compared to pure language models, such structure-aware generative approaches excel in multi-target optimization and structural hotspot preservation.

In recent years, nanobodies have attracted increasing attention as emerging recognition elements in electrochemical biosensors [56,57]. Nanobodies, also known as single-domain antibodies (VHHs), are antibody fragments derived from the heavy-chain antibodies of camelids, consisting of a single antigen-binding domain. Compared with conventional antibodies, nanobodies possess a smaller molecular weight, superior stability, and enhanced resistance to denaturation and are readily amenable to genetic engineering. The AbNatiV model developed by Ramon et al. enables the quantification of antibody/nanobody “nativeness” and predicts immunogenicity through residue-level mapping, thereby facilitating automated humanization and markedly improving affinity and stability [58]. Similarly, Deng et al. introduced the DeepNano framework, which leverages specialized NAI datasets and prompt-driven protein language models to outperform general algorithms in binding site prediction and virtual screening [59]. Collectively, these approaches enhance molecular usability assessment and binding specificity prediction, thereby accelerating the design and screening of nanobodies. Nevertheless, in the context of electrochemical pathogen detection, challenges persist due to limited data coverage, poor environmental adaptability, and the absence of experimental–computational closed-loop systems, underscoring the need for the integration of real-world samples and interface optimization to achieve practical performance translation.

Comparable methodologies are being extended to non-protein recognition molecules. Douaki et al., for instance, developed a “smart SELEX” framework by integrating CNN into aptamer screening workflows. By extracting interaction fingerprints between candidate sequences and targets, the model compressed vast sequence libraries into high-affinity subsets with reduced screening iterations (Figure 3B), outperforming traditional SELEX in terms of both sensitivity and selectivity during ammonia detection [60].

For enzymatic sequences, annotation has evolved beyond homology-based approaches toward contrastive learning strategies. The CLEAN model exemplifies this shift by constructing positive–negative enzyme pairs with divergent structures but similar functions. Through maximizing representational proximity in embedding space, CLEAN achieves highly accurate functional annotation, particularly for under-characterized or promiscuous enzyme families, thereby advancing resource discovery and classification efforts [61].

Once preliminary structural models or high-affinity candidates are identified, further refinement must often balance catalytic efficiency, binding strength, specificity, and conformational robustness. AI facilitates this via quantitative structure–function models coupled with optimization algorithms such as GA and Bayesian Optimization. For instance, Tuta-Navajas et al. embedded fitness functions into GA to invert enzyme kinetic parameters (e.g., *k*_cat_, *K*_m_). Their model accurately predicted rate constants for each step in enzymatic pathways, achieving over 99% agreement with experimental data across complex substrate–enzyme systems [62]. This method circumvents the need for extensive wet-lab kinetics, offering promising applications in cascade pathway design and heterologous expression systems.

In multi-objective scenarios, Bachas et al. proposed augmenting affinity prediction with a “naturalness” metric, defined as the sequence’s conformity to native immunoglobulin language distributions. This metric approximates immunogenicity and conformational feasibility. They demonstrated that affinity-optimized sequences with low naturalness often exhibit poor expression or stability. By simultaneously optimizing both metrics via GA, they enhanced the developability of candidate antibodies [63].

Similar strategies have been employed in nucleic acid modeling. Li et al. developed a framework combining pseudo-amino acid encoding, feature incremental selection, and Random Forest classification to predict aptamer–target binding interactions. The resulting model surpassed 77% accuracy, emphasizing feature coupling between sequence structure and binding-site properties rather than traditional reliance on binding energy alone [64]. AI’s principal advantage here lies in its ability to provide a global perspective for high-dimensional, multi-objective optimization, mitigating local optima and expediting experimental validation through strategic search.

Despite robust design, recognition molecules may exhibit diminished performance under complex real-world conditions, such as pH fluctuations, temperature shifts, ionic interference, or electrode surface interactions. Yu et al. proposed the UniKP framework, which combines language models and substrate fingerprints with environmental variables using a dual-layer architecture. The base layer predicts catalytic activity from molecular descriptors, while the meta-layer modulates outputs based on contextual factors like pH and temperature. This robust prediction mechanism successfully identified thermostable and acid-resistant tyrosinase variants through directed evolution (Figure 3C) [65].

For nucleic acids, environmental adaptability hinges on conformational flexibility and target specificity [66,67]. AI models can predict secondary structure folding under varying salt concentrations or electric fields, guiding the strategic incorporation of locked nucleic acids (LNA) or methylation modifications to prevent instability or off-target binding [68,69]. Furthermore, recognition elements must often function at abiotic interfaces such as electrodes or micro/nanostructured surfaces. Future AI developments will increasingly integrate interfacial physics, such as molecular dynamics and multiscale modeling, to predict conformational compatibility and non-specific adsorption at electrode–molecule boundaries, ultimately enabling true “end-to-end” environmental adaptability in sensor design.

### 3.2. Algorithmic Regulation of Sensing Materials and Sensor Parameters

The overall performance of electrochemical sensors is determined not only by the properties of their biorecognition elements but also by the composition and structural characteristics of the sensing materials, as well as the precise configuration of sensor parameters. Critical material attributes, such as conductivity and porosity, together with operational parameters, exert profound influences on signal transduction efficiency, stability, and sensitivity [70,71,72,73]. Artificial intelligence (AI), particularly machine learning (ML) and optimization algorithms, has been progressively integrated into both the development of sensing materials and the fine-tuning of electrochemical sensor parameters. This AI-driven paradigm enables the construction of intricate mapping relationships between material structures/parameters and sensor performance, thereby facilitating targeted optimization. Such an approach markedly enhances both the efficiency and controllability of sensor design.

#### 3.2.1. Algorithmic Optimization of Sensing Materials

The application of artificial intelligence in materials design has evolved from early-stage performance prediction to more sophisticated strategies involving structural elucidation and compositional optimization. Within the domain of electrochemical sensors, the roles of conductivity and porosity differ across components: for electrode substrates (e.g., gold electrodes, glassy carbon electrodes), high conductivity is fundamental to ensuring efficient electron transport; whereas for electrode modifiers (e.g., metal–organic framework (MOF)-derived materials and nanocomposites), conductivity and porosity jointly constitute the performance-defining attributes—conductivity enhances interfacial electron transfer, while porosity expands the specific surface area and facilitates mass transport, thereby significantly improving the utilization of active sites.

**Figure 4:**
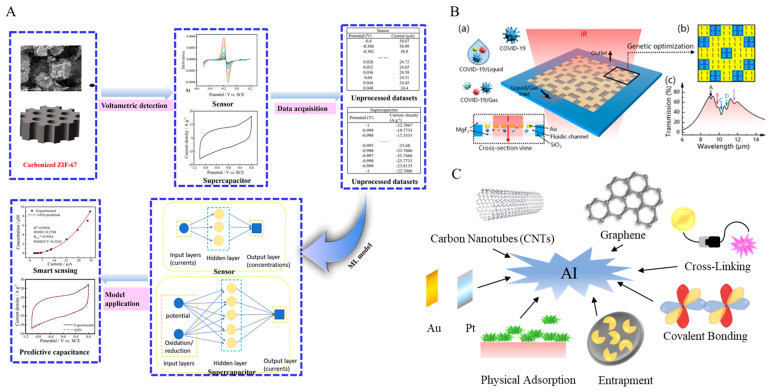
Applications of AI in the Development of Electrochemical Sensors. (**A**) The manufacturing and application of ANN for sensors and supercapacitors. Reproduced with permission from reference [74]. (**B**) Genetic Algorithm-based optimization of plasmonic biosensors for COVID-19 detection. (**a**) Transmission spectra of the metal structure covered with different thicknesses of the COVID-19 molecular layer. The thickness of the COVID-19 molecular layer varies from 0 to 110 nm. (**b**) Corresponding relative signal spectrum. (**c**) Relative signal strength of the five fingerprint peaks (A–E) of COVID-19 molecules varies with thickness. Reproduced with permission from reference [79]. (**C**) AI-Based Combination Optimization.

Lu et al. investigated carbonized MOF (C-ZIF-67) as an electrode modifier, employing an Artificial Neural Network (ANN) model to establish complex correlations between the microstructural features of C-ZIF-67 and its sensitivity toward niclosamide (NA) detection (Figure 4A). Beyond achieving reverse performance prediction and structural recommendation within high-dimensional spaces, the model innovatively incorporated derivative-processed voltammetric data, effectively minimizing human reading errors and substantially improving response sensitivity at low concentrations. Complementary theoretical calculations of adsorption and binding energies further guided material optimization. The experimental outcomes were striking: the optimized sensor exhibited a 196.6-fold enhancement in response compared with the bare glassy carbon electrode (GCE), with an exceptionally broad linear range (1 nM–9 μM) and an ultra-low detection limit (0.3 nM). Remarkably, the ANN model also successfully predicted the performance of C-ZIF-67 in supercapacitors (336.67 F/g at 2 A/g) with controllable error, thereby underscoring the potential of machine learning in developing multifunctional electrode materials [53]. The superior properties of C-ZIF-67 derive the following from its unique architecture: carbonization produced a graphitic carbon framework that significantly enhanced conductivity, while the periodic channels inherited from the MOF precursor conferred high porosity [74].

Kavya et al. in contrast, explored a composite of gold nanofibers integrated with a chromium-based MOF (MIL-101(Cr)-NH_2_). They constructed a modeling framework integrating multiple algorithms—including Random Forest, Support Vector Machines, and Gradient Boosted Trees—to optimize its performance for caffeic acid (CA) detection. The composite material benefited from its distinctive 1D/2D architecture: gold nanofibers of approximately 12 nm diameter provided highly conductive pathways that accelerated electron transfer, while their interwoven three-dimensional network created abundant secondary pores, substantially enhancing the capture efficiency of CA molecules. The study not only employed microscopic parameters for response prediction but also pioneered the use of Convolutional Neural Networks (CNNs) coupled with computer vision to directly extract conductivity-related features from pore structure images. The predictive results from multiple algorithms aligned closely with experimental data. Empirical tests demonstrated a wide linear range (0.5–100 μM), ultra-low detection limit (0.011 μM), and high sensitivity (2.53 µA/µM/cm^2^). Furthermore, the sensor exhibited outstanding anti-interference capacity and reliability in spiked recovery experiments with real samples such as coffee powder and red wine [54]. In this case, the gold nanofibers simultaneously fulfilled the dual roles of conductivity enhancers and contributors to secondary porosity [75].

These case studies clearly demonstrate the formidable potential of AI algorithms in optimizing multicomponent, multifunctional electrode modification materials. Whether in structurally intricate single-derived materials such as C-ZIF-67 or well-defined composites like AuNF/MOF hybrids, performance attributes—conductivity, porosity, and selectivity—are often governed by the synergistic interplay of distinct structural features. Leveraging powerful data processing, pattern recognition, and predictive optimization capabilities, AI can efficiently explore such complex design spaces, enabling precise, targeted performance enhancement and thereby accelerating the development of high-performance electrochemical sensing platforms.

Of particular significance is the translational value of these AI optimization strategies beyond the studied targets, namely, niclosamide and caffeic acid, toward foodborne pathogen detection. The underlying logic aligns closely with the critical demands of pathogen sensing, which center on two primary challenges: ultra-trace analyte capture (necessitating exceptional sensitivity and low detection limits) and interference elimination in complex matrices (requiring strong anti-fouling and selectivity). Lu’s work, which achieved a 196.6-fold response enhancement and a detection limit of 0.3 nM through AI-optimized conductivity and porosity, directly informs the capture and amplification of trace pathogen biomarkers such as toxins or nucleic acid fragments [74]. Similarly, Kaveia’s strategy of tailoring pore architectures and deploying ensemble learning to suppress nonspecific adsorption in challenging matrices offers direct guidance for selective recognition of pathogens in real food samples [75]. Such an end-to-end optimization framework—spanning material design, performance engineering, and application context—presents a transferable paradigm for the efficient construction of robust, AI-enhanced sensing platforms targeting foodborne pathogens.

#### 3.2.2. Algorithmic Regulation of Sensor Parameters

The performance of electrochemical sensors is governed not only by the intrinsic properties of the materials but also by a suite of critical parameters. These include material composition (e.g., concentration and ratios of components), fabrication and testing conditions (e.g., reaction cycles, processing durations), environmental factors (e.g., pH, temperature), and structural characteristics [76]. The interactions among these variables are often highly nonlinear, rendering traditional single-factor experiments insufficient for deciphering such complex coupling effects. AI-driven optimization methods, however, provide a powerful and efficient means of addressing this challenge.

Aydin Imani et al. developed an electrochemical nanobiosensor based on gold nanorods (GNRs) and graphene oxide (GO) and integrated Artificial Neural Networks (ANNs) with Genetic Algorithms (GAs) to achieve multivariable synergistic optimization. Eight influential parameters—including GO concentration, GNR concentration, probe concentration, probe incubation time, mercaptohexanol (MCH) treatment time, hybridization time, Oracet Blue (OB) concentration, and OB incubation time—were used as model inputs. The trained model achieved a prediction accuracy of 96.91%, with a mean absolute percentage error (MAPE) of only 5.509%. More importantly, the optimal parameter set identified by the GA outperformed even the best manually optimized laboratory conditions [77]. This approach effectively addressed the challenge of navigating the vast, nonlinear parameter space inherent in nanocomposite systems, thereby providing robust theoretical support for multivariable optimization.

In the field of molecularly imprinted polymers (MIPs), template recognition performance—typically quantified by the imprinting factor (IF)—is highly sensitive to synthesis conditions. Traditional experimental optimization not only requires substantial reagent consumption but is also labor-intensive and time-consuming. Yarahmadi et al. sought to address this limitation by employing machine learning to predict IF values. They investigated a broad range of input features, including pH, template type, monomer type, solvent category, and partition coefficients of both MIPs (KMIP) and non-imprinted polymers (KNIP), while benchmarking the predictive performance of multiple algorithms, including classification and regression trees, support vector regression, k-nearest neighbors, gradient boosting (GB), Random Forests, and extremely randomized trees. Among these, gradient boosting achieved the best results, with an R^2^ of 0.871 and remarkably low mean absolute error (MAE, −0.982) and mean squared error (MSE, −2.303), substantially outperforming conventional empirical optimization methods [78]. This data-driven approach not only reduced the experimental cost of MIP synthesis but also elucidated the nuanced relationships between synthesis parameters and imprinting performance.

AI-driven optimization has also extended to plasmonic-enhanced sensing systems. Li et al. developed a surface-enhanced infrared absorption (SEIRA) plasmonic biosensor for COVID-19 detection, in which Genetic Algorithms were employed to intelligently optimize key nanostructural parameters. The resulting device exhibited ultrahigh sensitivity (1.66%/nm), a broad detection range, and robust adaptability to both gaseous and liquid environments. Leveraging the molecular fingerprinting capability of infrared spectroscopy, the sensor further demonstrated potential for variant strain screening (Figure 4B) [79]. Such holistic optimization—from physical structure to operational parameters—signifies a paradigm shift in which AI has moved beyond a supporting role to become a central driving force in sensor design. Particularly during emergent public health crises such as the COVID-19 pandemic, the rapid response and efficient optimization enabled by AI provide critical support for real-time biosensing technologies.

Equally critical is the immobilization of biorecognition elements on sensor surfaces, a process that directly influences signal stability and reproducibility. Whether through physical adsorption, covalent bonding, or encapsulation, striking an optimal balance among sensitivity, stability, and reversibility remains essential. Parameter settings, such as pH, incubation time, and temperature, are not only intrinsically complex but also closely interdependent with material type and interfacial chemistry. By integrating AI methods such as Genetic Algorithms and Bayesian Optimization, systems can autonomously identify the most effective immobilization strategies from a pool of candidates while adaptively tuning reaction conditions to maximize binding efficiency and signal stability. Such optimization is particularly valuable for complex systems requiring the integration of multiple immobilization strategies, offering a repeatable and efficient pathway for addressing sophisticated immobilization demands (Figure 4C).

### 3.3. AI-Driven Analysis and Optimization of Electrochemical Signals

The performance of electrochemical sensing systems hinges not only on the design of recognition elements and functional materials at the front end but also on the intelligent interpretation of complex signal outputs. In the context of foodborne pathogen detection, conventional methodologies exhibit clear limitations in managing high-dimensional data, resisting environmental perturbations, and deconvoluting multi-component signals. The integration of AI has brought transformative advancements to this critical domain. By employing ML models for feature extraction, error correction, and component identification, AI effectively addresses challenges such as signal distortion, data complexity, and interference from coexisting species—thereby offering a more intelligent and robust pathway for food safety monitoring [80].

#### 3.3.1. High-Dimensional Signal Modeling

Electrochemical data are often characterized by high dimensionality, pronounced nonlinearity, and susceptibility to noise interference. Traditional approaches that rely on empirical feature extraction and heuristic modeling struggle to maintain efficiency and accuracy when confronted with large-scale, multivariable datasets [81]. Gundry et al. applied ML techniques to analyze experimental and simulated data from large-amplitude alternating current voltammetry, significantly enhancing both the precision of electrochemical parameter extraction and modeling efficiency [82]. This work marks a paradigm shift from conventional modeling to intelligent signal interpretation.

To address the nonlinear and high-dimensional nature of electrochemical signals, dimensionality reduction techniques such as PCA and t-SNE have been widely employed to eliminate redundancy while preserving critical reactive features. When combined with supervised learning methods such as SVM and RF, these tools enable automated feature selection and high-accuracy predictive modeling. Further advances are exemplified by the Sure Independence Screening and Sparsifying Operator (SISSO), which extracts interpretable physical descriptors from complex variable sets, offering both strong predictive performance and theoretical clarity [83].

**Figure 5:**
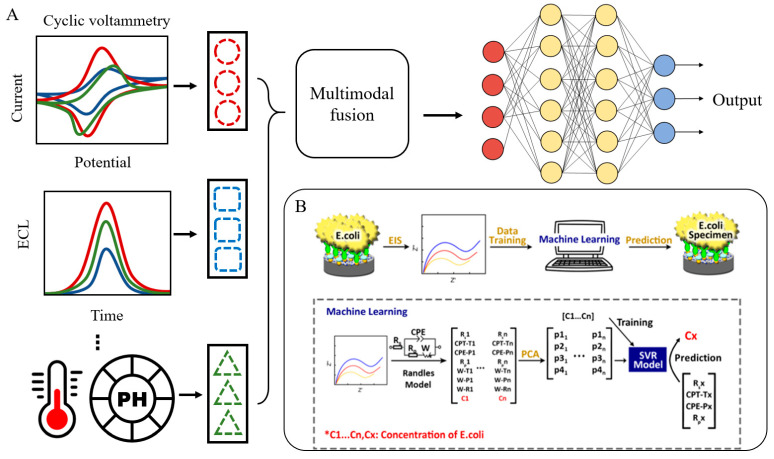
AI-enabled optimization of electrochemical signal processing. (**A**) Multimodal fusion of electrochemical and environmental data. (**B**) Machine learning-based signal processing workflow. Reproduced with permission from reference [84].

In a practical application, Xu et al. integrated ML with EIS to construct an intelligent detection model for the quantitative identification of *Escherichia coli*. By leveraging PCA and SVR, their method extracted multiple impedance parameters from EIS data and automatically mapped these parameters to bacterial concentrations (Figure 5B), significantly enhancing detection accuracy [84]. This study underscores the potential of combining multi-parametric impedance information with AI algorithms and provides a foundational framework for multimodal data fusion strategies.

Aligned with this trajectory, contemporary research increasingly emphasizes the integrated analysis of multimodal electrochemical data to improve sensing throughput and system robustness. This approach synthesizes data from various measurement modalities, such as CV and electrochemiluminescence (ECL) imaging, to extract complementary features, thereby overcoming the informational limitations of single-source signals and enabling more comprehensive data interpretation and performance enhancement (Figure 5A). For instance, Ccopa Rivera et al. developed a smartphone-based ECL sensor by simultaneously capturing ECL images and amperometric curves and analyzing the fused data using AI algorithms. Their model, combining RF and Feedforward Neural Network (FNN) architectures, achieved high-precision prediction of the luminophore Ru(bpy)_3_^2+^ concentration, with coefficients of determination reaching R^2^ = 0.99 for RF and R^2^ = 0.96 for FNN within the experimental detection range—demonstrating the remarkable efficacy of AI in modeling multimodal datasets. (In this context, “ECL images” specifically refer to spatially resolved imaging of luminescent signals triggered by electrochemical reactions. The underlying signal fundamentally originates from electrochemical processes at the electrode interface, representing an extension of conventional ECL techniques into the dimension of spatial resolution.) [85].

#### 3.3.2. Signal Compensation and Calibration

In electrochemical sensing systems, the accuracy of detection signals is influenced not only by the intrinsic performance of electrode materials but also by a multitude of external perturbations. Among these, the surface condition of the electrode and matrix effects from sample composition constitute two primary sources of interference.

During routine use, electrodes are prone to contamination, such as the adsorption of impurities, deposition of reaction byproducts, or interactions with electrolyte constituents, which degrades their electrochemical activity and results in signal attenuation and distortion. This phenomenon, commonly referred to as electrode fouling [86], undermines detection accuracy. While traditional approaches such as linear calibration and the method of standard additions offer partial correction, they often fall short under highly variable or interference-rich conditions and suffer from operational complexity and limited adaptability. To enhance the robustness of sensing systems under such challenging scenarios, recent studies have increasingly turned to ML techniques for modeling and compensating signal fluctuations.

For instance, Aiassa et al. employed a nonlinear modeling strategy based on Support Vector Classification (SVC) to correct the detection responses of propofol in complex matrices. Their findings demonstrated that models utilizing Gaussian Radial Basis Function (RBF) kernels achieved superior performance in compensating for electrode fouling, reaching a classification accuracy of 90.6%—markedly outperforming traditional linear models [87]. This highlights the capacity of nonlinear ML algorithms to effectively mitigate non-idealities in sensing signals, providing a more robust framework for continuous monitoring applications.

Beyond surface-related factors, matrix effects represent another critical source of signal distortion [88]. These effects arise when coexisting substances within the sample interfere with the accurate quantification of target analytes, frequently resulting in significant analytical bias. Conventional remedies such as the standard addition method rely on exogenous spiking, which is labor-intensive, inefficient, and ill-suited to dynamically fluctuating sample environments. To address these limitations, Jawaid et al. developed an intelligent sensor system based on ANN, which leveraged DPV data modeling to achieve high-precision detection of tetrabromobisphenol A (TBPA) in river water. Their study underscores the potential of ML in environmental signal correction and adaptive calibration, enabling sensors to maintain stable performance amidst environmental variability and ensuring consistent and accurate detection outcomes [89].

Notably, AI models possess the capability for retraining and transfer learning, allowing them to dynamically adapt to samples of diverse origins and environmental conditions. Exploiting this capacity, electrochemical sensors can sustain performance stability even in the face of electrode degradation or complex sample matrices, while also enabling autonomous compensation without reliance on external standards. Such advancements are steering sensing systems from static calibration schemes toward intelligent, dynamic correction paradigms—thereby laying a robust foundation for the development of high-throughput, high-accuracy food safety monitoring platforms tailored to real-world conditions.

#### 3.3.3. Multicomponent Detection

In recent years, the demand for electrochemical sensors in the analysis of complex samples has surged, rendering their capacity for multicomponent detection a pivotal metric in evaluating system performance. Such capability hinges on the differentiation of electrochemical responses, such as redox potentials and peak currents, among individual analytes. However, the coexistence of multiple species within a sample often results in overlapping signals and cross-interference, significantly undermining detection accuracy and specificity. Consequently, enhancing the resolution of electrochemical systems in multicomponent contexts has emerged as a focal point of contemporary research.

For example, Gu et al. employed an ANN model to jointly analyze signals derived from linear sweep voltammetry (LSV) and DPV, enabling the simultaneous quantification of caffeine (CAF) and chlorogenic acid (CGA). By extracting key features from the voltammograms and optimizing the structure of the training dataset, the model demonstrated accurate concentration predictions under conditions of signal overlap, thereby validating the efficacy of ANN in multicomponent modeling and signal deconvolution [90].

In more complex matrices, Bonet-San-Emeterio et al. integrated electrochemically reduced graphene oxide (ERGO)-modified electrodes with ANN modeling to achieve efficient discrimination and the quantification of dopamine, serotonin, and interfering substances such as ascorbic acid and uric acid. This approach effectively resolved signal overlap and exhibited exceptional sensitivity and accuracy in practical applications [91]. Similarly, Torrecilla et al. developed an amperometric biosensor based on a gold nanoparticle-enzyme electrode and utilized an ANN to process the electrochemical data for the simultaneous quantification of glucose and its common interferents. Without the need for any prior signal preprocessing, the ANN achieved correlation coefficients exceeding 0.99 and average prediction errors below 1.7%, underscoring its robustness in handling complex electrochemical signals [92].

Addressing real-world detection challenges, Lee et al. further designed an ML-enabled electrochemical aptasensor, enhanced with a gold nanoflower-modified interface to boost sensitivity, and applied the Least Squares Boosting (LSBoost) algorithm for data modeling and error correction. This system exhibited exceptional anti-interference capability in the simultaneous detection of di (2-ethylhexyl) phthalate (DEHP) and bisphenol A (BPA) in river water under varying pH conditions, achieving detection limits at the picomolar level [93].

Collectively, ML-driven multicomponent electrochemical sensing has achieved notable success in the analysis of small molecules. With continued technological advancements, such approaches are poised to extend into more intricate domains such as the rapid identification of foodborne pathogens, offering intelligent, high-throughput solutions for on-site food safety screening and propelling detection technologies toward greater efficiency and precision.

### 3.4. AI-Internet of Things (IoT) Integrated Sensing Platforms for On-Site Applications

As electrochemical biosensors transition toward point-of-care (PoC) diagnostics and large-scale deployment, system intelligence has become a critical direction for technological evolution. Conventional sensors, heavily reliant on laboratory operations, often fail to meet the diverse demands of real-world applications, such as operation in non-specialist environments, high-frequency monitoring, and remote responsiveness. The integration of the IoT with AI is forging a new paradigm of intelligent sensing systems equipped with real-time data acquisition, remote control, and autonomous decision-making capabilities, thereby providing a foundational architecture for next-generation PoC platforms [94].

A typical IoT-based sensing architecture comprises sensor nodes, wireless communication modules, and data processing units. The sensor nodes detect target analytes and convert signals, which are then transmitted via wireless protocols such as Wi-Fi, Bluetooth, or LoRa to local servers or cloud platforms. Backend systems subsequently perform data analysis and feedback control [95,96,97]. This process significantly reduces the need for user intervention, allowing PoC devices to operate autonomously over extended periods in remote settings, with capabilities for dynamic parameter adjustment, self-diagnosis, and real-time data synchronization.

More importantly, IoT systems ensure a stable and continuous flow of high-quality data, establishing a robust foundation for the deployment and refinement of AI models [98]. Environmental variables, such as temperature, humidity, and electrode condition, alongside analytical signals, can be recorded in real time, supporting AI-driven adaptive calibration, fault detection, and trend forecasting. Consequently, PoC platforms gain the capacity to “detect, learn, and optimize” simultaneously.

**Figure 6:**
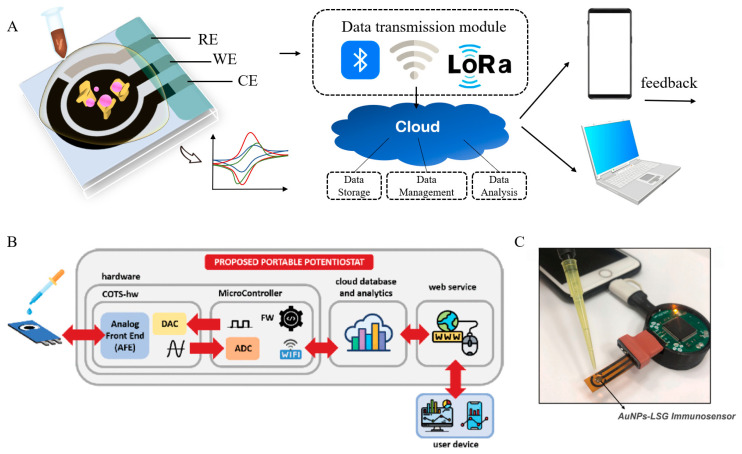
AI-integrated Internet of Things (IoT)-enabled electrochemical biosensing systems. (**A**) IoT-coupled electrochemical biosensors. (**B**) Architecture of a portable electrochemical workstation. Reproduced with permission from reference [100]. (**C**) USB-C connected portable point-of-care (PoC) device interfacing with a smartphone. Reproduced with permission from reference [102].

On this foundation, the deep integration of AI and IoT is catalyzing the transformation of electrochemical PoC devices from reactive terminals to intelligent decision-making nodes. As illustrated in Figure 6A, the intelligent sensing system centers on a three-electrode configuration, wherein generated signals are transmitted via IoT modules to the cloud. AI models then perform classification or regression analyses, with the outcomes instantly relayed to mobile terminals, thereby achieving a closed-loop of detection, analysis, and feedback [99].

Bianchi et al. developed a portable electrochemical detection device that exemplifies the implementation of this architecture. The device is equipped with a built-in Wi-Fi module, enabling direct cloud transmission of collected data, which is then classified via an SVM model and displayed through a web interface. Requiring no dedicated app, the system simplifies user interaction while delivering analytical performance comparable to standard laboratory instruments. The platform is particularly suitable for medical PoC scenarios, where clinicians can remotely access results and initiate treatment strategies (Figure 6B) [100].

The advantages of such platforms become even more pronounced during public health emergencies. Kaushik et al. engineered a portable system integrating miniaturized electrochemical biosensors, nanomaterial-based functional layers, and IoT modules to detect SARS-CoV-2 antigens. Powered by AI analytics, the system enabled real-time data uploading and contributed to epidemiological surveillance, early screening, and individualized intervention [101]. Building on this concept, Beduk et al. developed a PoC device capable of differentiating between SARS-CoV-2 variants (e.g., Alpha, Beta, Delta), incorporating gold nanoparticles and ACE2-based recognition with a dense neural network to enhance classification accuracy. Detection was completed in under one minute (Figure 6C) [102].

AI-IoT-PoC systems have also been extended to the rapid detection of pathogenic bacteria. One study developed an intelligent diagnostic platform utilizing a wireless microscope and microfluidic chip. Images were uploaded via a smartphone and analyzed using an XGBoost model to identify 10 common bacterial species, including *Escherichia coli*, *Salmonella*, and *Staphylococcus aureus*, achieving an accuracy of 91.67% within 30 min—ideal for rapid screening in water and dairy samples [103].

The potential of AI-IoT-PoC systems in monitoring across the entire food supply chain is gradually being realized. From farm to table, smart sensors can enable real-time monitoring of environmental parameters, microbial contamination, and transportation conditions. For instance, in the processing stage, the system can detect heat and humidity anomalies and adjust production parameters accordingly; in cold-chain logistics, it can trace temperature fluctuations and issue alerts in case of transportation irregularities, thereby enhancing traceability and safety. This end-to-end intelligent control model is reshaping the food safety infrastructure, enabling unmanned detection, proactive warning systems, and region-specific responses. Table 2 systematically outlines the optimization task categorization, corresponding methodologies, and technical advantages of AI-enabled optimization in intelligent electrochemical biosensing systems across various modules, supplemented with representative research cases.

## 4. Conclusions and Future Perspectives

The integration of artificial intelligence (AI) is profoundly reshaping both the design logic and application paradigm of electrochemical biosensing systems. Particularly in foodborne pathogen detection, AI has demonstrated substantial potential in enhancing sensitivity, selectivity, and system intelligence. Through AI-driven molecular design, material optimization, signal interpretation, and system integration, biosensors are evolving from laboratory prototypes into deployable platforms characterized by higher levels of automation, throughput, and environmental adaptability.

However, despite this progress, the field currently stands at a critical transitional juncture—shifting from methodological exploration to practical deployment—where numerous challenges remain unresolved. Data-related limitations are especially pronounced: real-world food matrices are compositionally complex and highly variable, with chemical additives and environmental fluctuations (e.g., temperature, humidity) introducing significant signal interference. Current AI models are often trained on data from controlled laboratory settings, which fail to capture such variability, leading to limited generalizability and reduced performance in field scenarios. Moreover, the uneven distribution of training data—overrepresenting certain conditions while lacking samples from rare or extreme scenarios—further weakens model robustness. For instance, pesticide residue detection across fruits of different origins is often confounded by intrinsic compositional differences that existing models cannot accommodate.

In addition, many AI models are built on statistical correlations without integrating underlying physical or chemical mechanisms. As a result, predictions, such as analyte concentrations, often lack interpretability grounded in electrochemical principles like electron transfer dynamics or interfacial adsorption–desorption kinetics. Such “black-box” approaches hinder adoption in high-stakes domains like medical diagnostics or food safety, where decision transparency and scientific justification are essential.

From a practical standpoint, trade-offs among key sensing metrics, such as sensitivity, response time, and cost, pose further design challenges. Ultra-sensitive systems often require elaborate detection structures or expensive materials, which compromise speed and cost-effectiveness. Current AI-based optimization methods frequently target single objectives, lacking the capability to coordinate system-level performance across multiple, sometimes conflicting, dimensions.

To address these bottlenecks, the evolution of AI-enabled sensing capabilities must proceed through system-level, cross-domain co-optimization. Mechanism-informed modeling approaches that integrate physical principles into learning frameworks can improve model interpretability and cross-platform transferability. Structured, high-quality, and multimodal datasets will be essential for robust signal modeling, enabling fine-grained decomposition and compensation of multicomponent interference. Furthermore, adaptive AI architectures equipped with self-calibration, online updating, and fault prediction functionalities will support long-term sensor reliability in dynamic environments, while multi-objective optimization algorithms will enhance overall system usability and scalability. To this end, Table 3 summarizes key AI applications across sensing subsystems, outlines prevailing technical bottlenecks, and suggests prospective research directions.

Importantly, the convergence of AI and the Internet of Things (IoT) offers a foundation for achieving this systemic transformation. IoT-enabled biosensors can provide real-time environmental context, such as temperature, humidity, and sample origin, that AI models can leverage for dynamic correction and personalized adaptation. This deep integration facilitates a closed-loop workflow of “sensing–learning–responding–optimizing”, bridging the gap between static analysis and intelligent decision-making.

Ultimately, the AI-driven evolution of biosensors should not be confined to functional enhancement at the component level but rather be orchestrated as a co-evolution across molecular, material, signal, and system layers. Meeting the challenges of real-world deployment demands not only algorithmic advancement but also deep interdisciplinary collaboration, spanning materials science, bioengineering, data science, and embedded systems. In this context, intelligent biosensors are poised to move beyond their role as passive analytical devices to become active, decision-making nodes within a scalable, responsive food safety monitoring network.

## Figures and Tables

**Table 1:** Artificial Intelligence (AI) Methodologies and Their Functional Matching Across Key Modules of Electrochemical Biosensing Systems.

Sensing Module	AI Methodology	Distinctive Strengths	Concise Descriptions and Examples
Biorecognition Molecule Design	Protein language models, GNNs	Cross-structural modeling, label-free learning	Design high-performance antibodies, aptamers, or enzymes by optimizing sequence-structure-function relationships (e.g., ESM models for antibody affinity or CNN-based “smart SELEX” for aptamer screening).
Material Structure and Parameter Tuning	GA, Bayesian Optimization, RL	Global search, multi-objective trade-off	Enhance conductivity/stability by tuning parameters like nanoporous gold porosity or MOFs synthesis temperature.
Electrochemical Signal Modeling and Noise Reduction	ANN, SVM, RF, CNN	Nonlinear modeling, automatic feature extraction	Improve accuracy via RF for baseline drift correction or CNN for impedance feature extraction.
Environmental Compensation and Auto-Calibration	Few-Shot Learning, Transfer Learning, RBF Kernels	Anomaly modeling, strong adaptability	Compensate temperature/pH interference with RBF kernels or transfer learning.
Multimodal Data Fusion and Feedback Control	Multimodal Neural Networks, Edge Deep Learning	Data integration, low-latency processing	Enable real-time control by fusing electrochemical/spectral data with edge AI.

Note: GNNs: Graph Neural Networks; GA: Genetic Algorithms; RL: Reinforcement Learning; ANN: Artificial Neural Network; SVM: Support Vector Machine; RF: Random Forest; CNN: Convolutional Neural Network; RBF: Radial Basis Function.

**Table 2:** Task Categorization and Methodological Mapping of AI-Enabled Optimization in Intelligent Electrochemical Biosensing Systems.

Module Category	Optimization Subtask	AI Methodology	Technical Advantages	Representative Study
Molecular Design	Sequence Function Annotation	Contrastive Learning, ESM	No structural input required; suitable for data-scarce conditions	[54,61]
	Structure-Guided Optimization	AlphaFold + ESM-IF1, GNNs	Cross-target transferability; balanced binding affinity and stability	[55]
	Multi-Objective Property Tuning	GA, Bayesian Optimization	Simultaneous optimization of affinity and “nativeness”	[63]
	Non-Protein Molecule Design	CNN-SELEX, Feature Embedding	Sequence compression, cycle reduction, complex target adaptation	[60]
	Environmental Adaptation Modeling	Meta-Learning, Language Models + Knowledge Graphs	Integrates external conditions like pH and temperature	[65]
Materials and Parameters	Electrode Material Property Prediction	ANN, CNN, SVM	Image + structure modeling; high-dimensional performance mapping	[74]
	Conductive Material Structure Tuning	Bayesian Optimization, GNN	Material graph interpretation; inverse design	[75]
	Joint Optimization of Working Parameters	ANN + GA	Multi-parameter coupling; global optimization	[77]
	Plasmonic Structure Design	GA	Structure-signal co-modeling; detection limit enhancement	[79]
Signal Processing	High-Dimensional Signal Modeling	SVM, RF, CNN, PCA	Nonlinear modeling; data compression; variable selection	[82,84]
	Multimodal Data Fusion	Multimodal Deep Networks, Ensemble Learning	Joint modeling of image and current signals	[85]
	Signal Compensation/Correction	SVC, RBF Kernel, ANN	Nonlinear interference modeling; adaptive response tuning	[87]
	Matrix Effect Compensation	ANN, Few-Shot Learning	Environment adaptation without external standards	[89]
	Multicomponent Detection	ANN, Boosting Algorithms	Signal deconvolution; peak recognition; interference resolution	[90,91,92]
System Integration	Real-Time Data Upload and Control	IoT + Edge AI	Automated sensing; remote transmission; feedback loop	[100]
	Low-Power On-Site Deployment	Lightweight Neural Networks, Compressed Models	Extended monitoring; portable integration; resource efficiency	[101]
	Variant Recognition and Risk Forecasting	Deep Neural Networks	Multi-class classification; high-sensitivity screening	[102]

Note: ESM: Evolutionary Scale Modeling; PCA: Principal Component Analysis.

**Table 3:** Application Landscape and Research Outlook of AI in Electrochemical Biosensing Systems.

Optimization Module	Current AI Applications	Technical Bottlenecks
Recognition Molecule Design	Sequence modeling, functional prediction	Label scarcity
Electrode Material Optimization	Performance regression, structure generation	Insufficient experimental validation
Electrochemical Signal Analysis	Pattern recognition and classification	High feature interference
Response Parameter Prediction	Sensitivity/LOD modeling	Data imbalance
Multicomponent Detection	Signal deconvolution and discrimination	Peak overlap interference
Dynamic Adaptation	Noise modeling, self-calibration	Poor cross-scenario adaptability
Long-Term Stability Modeling	Degradation forecasting, fault monitoring	Lack of real-world condition data
Multi-Objective Optimization	Hyperparameter search, multi-task learning	Frequent performance trade-offs
System Integration	IoT connectivity and feedback control	Power/compute resource limitations

## Data Availability

No new data were created or analyzed in this study.

## References

[1] Foodborne Diseases Estimates. https://www.who.int/data/gho/data/themes/who-estimates-of-the-global-burden-of-foodborne-diseases.

[2] Shen Y., Xu L., Li Y. (2021). Biosensors for Rapid Detection of Salmonella in Food: A Review. Compr. Rev. Food Sci. Food Saf..

[3] Shih C.-M., Chang C.-L., Hsu M.-Y., Lin J.-Y., Kuan C.-M., Wang H.-K., Huang C.-T., Chung M.-C., Huang K.-C., Hsu C.-E. (2015). Paper-Based ELISA to Rapidly Detect *Escherichia coli*. Talanta.

[4] Alarcón B., Vicedo B., Aznar R. (2006). PCR-based Procedures for Detection and Quantification of Staphylococcus Aureus and Their Application in Food. J. Appl. Microbiol..

[5] Quintela I.A., Vasse T., Lin C.-S., Wu V.C.H. (2022). Advances, Applications, and Limitations of Portable and Rapid Detection Technologies for Routinely Encountered Foodborne Pathogens. Front. Microbiol..

[6] Castle L.M., Schuh D.A., Reynolds E.E., Furst A.L. (2021). Electrochemical Sensors to Detect Bacterial Foodborne Pathogens. ACS Sens..

[7] Kucherenko I.S., Soldatkin O.O., Dzyadevych S.V., Soldatkin A.P. (2020). Electrochemical Biosensors Based on Multienzyme Systems: Main Groups, Advantages and Limitations—A Review. Anal. Chim. Acta.

[8] Wu J., Liu H., Chen W., Ma B., Ju H. (2023). Device Integration of Electrochemical Biosensors. Nat. Rev. Bioeng..

[9] Kissel M., Schosland M., Töws J., Kalita D., Schneider Y., Kessler-Kühn J., Schröder S., Schubert J., Frankenberg F., Kwade A. (2025). Quantifying the Impact of Cathode Composite Mixing Quality on Active Mass Utilization and Reproducibility of Solid-State Battery Cells. Adv. Energy Mater..

[10] Zainul R., Rafika R., Hasanudin H., Laghari I.A., Hamdani D.M.H., Mapanta J., Handayana R.H., Delson D., Mandeli R.S., Putra H. (2024). Systematic Review of Electrochemical Stability and Performance Enhancement in Energy Storage and Analytical Applications. Asian J. Green Chem..

[11] Liu Z., Zeng Y., Tan J., Wang H., Zhu Y., Geng X., Guttmann P., Hou X., Yang Y., Xu Y. (2024). Revealing the Degradation Pathways of Layered Li-Rich Oxide Cathodes. Nat. Nanotechnol..

[12] Qazi R.A., Aman N., Ullah N., Jamila N., Bibi N. (2024). Recent Advancement for Enhanced *E. Coli* Detection in Electrochemical Biosensors. Microchem. J..

[13] Bacchu M.S., Ali M.R., Das S., Akter S., Sakamoto H., Suye S.-I., Rahman M.M., Campbell K., Khan M.Z.H. (2022). A DNA Functionalized Advanced Electrochemical Biosensor for Identification of the Foodborne Pathogen *Salmonella enterica* Serovar Typhi in Real Samples. Anal. Chim. Acta.

[14] Zolti O., Suganthan B., Maynard R., Asadi H., Locklin J., Ramasamy R.P. (2022). Electrochemical Biosensor for Rapid Detection of Listeria Monocytogenes. J. Electrochem. Soc..

[15] Wang B., Wang H., Lu X., Zheng X., Yang Z. (2023). Recent Advances in Electrochemical Biosensors for the Detection of Foodborne Pathogens: Current Perspective and Challenges. Foods.

[16] Veliscek Z., Perse L.S., Dominko R., Kelder E., Gaberscek M. (2015). Preparation, Characterisation and Optimisation of Lithium Battery Anodes Consisting of Silicon Synthesised Using Laser Assisted Chemical Vapour Pyrolysis. J. Power Sources.

[17] Zhu Z., Song H., Wang Y., Zhang Y.-H.P. (2022). Protein Engineering for Electrochemical Biosensors. Curr. Opin. Biotechnol..

[18] Bocan A., Siavash Moakhar R., del Real Mata C., Petkun M., De Iure-Grimmel T., Yedire S.G., Shieh H., Khorrami Jahromi A., Mahshid S.S., Mahshid S. (2025). Machine-Learning-Aided Advanced Electrochemical Biosensors. Adv. Mater..

[19] de Oliveira Filho J.I., Faleiros M.C., Ferreira D.C., Mani V., Salama K.N. (2023). Empowering Electrochemical Biosensors with AI: Overcoming Interference for Precise Dopamine Detection in Complex Samples. Adv. Intell. Syst..

[20] Bodkhe G.A., Kumar V., Li X., Pei S., Ma L., Kim M. (2025). Biosensors in Microbial Ecology: Revolutionizing Food Safety and Quality. Microorganisms.

[21] Cho I.-H., Kim D.H., Park S. (2020). Electrochemical Biosensors: Perspective on Functional Nanomaterials for on-Site Analysis. Biomater. Res..

[22] Silva N.F.D., Magalhães J.M.C.S., Freire C., Delerue-Matos C. (2018). Electrochemical Biosensors for *Salmonella*: State of the Art and Challenges in Food Safety Assessment. Biosens. Bioelectron..

[23] Cheng J., Liang T., Xie X.-Q., Feng Z., Meng L. (2024). A New Era of Antibody Discovery: An in-Depth Review of AI-Driven Approaches. Drug Discov. Today.

[24] Khan H., Jan Z., Ullah I., Alwabli A., Alharbi F., Habib S., Islam M., Shin B.-J., Lee M.Y., Koo J. (2024). A Deep Dive into AI Integration and Advanced Nanobiosensor Technologies for Enhanced Bacterial Infection Monitoring. Nanotechnol. Rev..

[25] Mishra P., Gupta D. Comparative Analysis of a Bioelectric Cell Biosensor Dataset Employing Machine Learning Classifiers for Reliable Listeria Monocytogenes Identification. Proceedings of the 2024 15th International Conference on Computing Communication and Networking Technologies (ICCCNT).

[26] Kumar Y., Kaur I., Mishra S. (2024). Foodborne Disease Symptoms, Diagnostics, and Predictions Using Artificial Intelligence-Based Learning Approaches: A Systematic Review. Arch. Comput. Methods Eng..

[27] Meskher H., Achi F., Ha S., Berregui B., Babanini F., Belkhalfa H. (2022). Sensitive rGO/MOF Based Electrochemical Sensor for Penta-Chlorophenol Detection: A Novel Artificial Neural Network (ANN) Application. Sens. Diagn..

[28] Choi H., Shin H., Cho H.U., Blaha C.D., Heien M.L., Oh Y., Lee K.H., Jang D.P. (2022). Neurochemical Concentration Prediction Using Deep Learning vs Principal Component Regression in Fast Scan Cyclic Voltammetry: A Comparison Study. ACS Chem. Neurosci..

[29] Chen J., Lu N., Wang X., Chen Y., Guo M., Xu Y. (2022). Time-Lapse Electrochemical Impedance Detection of Bacteria Proliferation for Accurate Antibiotic Evaluation. IEEE Sens. J..

[30] Du L., Thoma Y., Rodino F., Carrara S. (2024). Automatic Simulation of Electrochemical Sensors by Machine Learning for Drugs Quantification. Electrochim. Acta.

[31] Shin Y.-U., Yu S.I., Bae H., Jang A. (2025). Deep Learning-Enhanced Multi-Modal Modeling for Electrosorption Performance Prediction via Nyquist Plots. Environ. Res..

[32] Esmaeili F., Cassie E., Nguyen H.P.T., Plank N.O., Unsworth C.P., Wang A. (2023). Utilizing Deep Learning Algorithms for Signal Processing in Electrochemical Biosensors: From Data Augmentation to Detection and Quantification of Chemicals of Interest. Bioengineering.

[33] Chen Y., Chen X., Xu A., Sun Q., Peng X. (2022). A Hybrid CNN-Transformer Model for Ozone Concentration Prediction. Air Qual. Atmos. Health.

[34] Chandra A., Tünnermann L., Löfstedt T., Gratz R. (2023). Transformer-Based Deep Learning for Predicting Protein Properties in the Life Sciences. eLife.

[35] Hu Y., Sharma A., Dhiman G., Shabaz M. (2021). The Identification Nanoparticle Sensor Using Back Propagation Neural Network Optimized by Genetic Algorithm. J. Sens..

[36] Vakilian K.A. (2022). Optimization Methods Can Increase the Durability of Smart Electrochemical Biosensors. Proceedings of the 2022 8th Iranian Conference on Signal Processing and Intelligent Systems (ICSPIS).

[37] O’Sullivan E.J. (2024). Bayesian Optimization Strategies for Electrochemical Technology Development. Proceedings of the Electrochemical Society Meeting Abstracts PRiME2024.

[38] Cheon M., Byun H., Lee J.H. (2024). Non-Myopic Bayesian Optimization Using Model-Free Reinforcement Learning and Its Application to Optimization in Electrochemistry. Comput. Chem. Eng..

[39] Ferruz N., Höcker B. (2022). Controllable Protein Design with Language Models. Nat. Mach. Intell..

[40] Olayo-Alarcon R., Amstalden M.K., Zannoni A., Bajramovic M., Sharma C.M., Brochado A.R., Rezaei M., Müller C.L. (2025). Pre-Trained Molecular Representations Enable Antimicrobial Discovery. Nat. Commun..

[41] Briceno-Mena L.A., Romagnoli J.A., Arges C.G. (2022). PemNet: A Transfer Learning-Based Modeling Approach of High-Temperature Polymer Electrolyte Membrane Electrochemical Systems. Ind. Eng. Chem. Res..

[42] Jiao J. The Application of Transfer Learning Models in the Construction of Microbial Intelligent Monitoring and Identification System. Proceedings of the 2024 International Conference on Machine Intelligence and Digital Applications.

[43] Murugan K., Gopalakrishnan K., Sakthivel K., Subramanian S., Li I.-C., Lee Y.-Y., Chiu T.-W., Chang-Chien G.-P. (2024). Machine Learning-Driven Advances in Electrochemical Sensing: A Horizon Scan. J. Electrochem. Soc..

[44] Morsch F., Umasankar I.L., Moreta L.S., Latawa P., Lange D.B., Wengel J., Konjen H., Code C. (2023). AptaBERT: Predicting Aptamer Binding Interactions. bioRxiv.

[45] Selvam R., Lim I.H.Y., Lewis J.C., Lim C.H., Yap M.K.K., Tan H.S. (2023). Selecting Antibacterial Aptamers against the BamA Protein in Pseudomonas Aeruginosa by Incorporating Genetic Algorithm to Optimise Computational Screening Method. Sci. Rep..

[46] Roy T.S., Roy J.K., Mandal N. (2023). Conv-Random Forest-Based IoT: A Deep Learning Model Based on CNN and Random Forest for Classification and Analysis of Valvular Heart Diseases. IEEE Open J. Instrum. Meas..

[47] Khozeimeh F., Sharifrazi D., Izadi N.H., Joloudari J.H., Shoeibi A., Alizadehsani R., Tartibi M., Hussain S., Sani Z.A., Khodatars M. (2022). RF-CNN-F: Random Forest with Convolutional Neural Network Features for Coronary Artery Disease Diagnosis Based on Cardiac Magnetic Resonance. Sci. Rep..

[48] Tuerk C., Gold L. (1990). Systematic Evolution of Ligands by Exponential Enrichment: RNA Ligands to Bacteriophage T4 DNA Polymerase. Science.

[49] Arnold F.H. (2017). Directed Evolution: Bringing New Chemistry to Life. Angew. Chem. (Int. Ed. Engl.).

[50] Hardy D., Bill R.M., Jawhari A., Rothnie A.J. (2016). Overcoming Bottlenecks in the Membrane Protein Structural Biology Pipeline. Biochem. Soc. Trans..

[51] Wüthrich K. (2003). NMR Studies of Structure and Function of Biological Macromolecules. Biosci. Rep..

[52] Jumper J., Evans R., Pritzel A., Green T., Figurnov M., Ronneberger O., Tunyasuvunakool K., Bates R., Žídek A., Potapenko A. (2021). Highly Accurate Protein Structure Prediction with AlphaFold. Nature.

[53] Meier J., Rao R., Verkuil R., Liu J., Sercu T., Rives A. (2021). Language Models Enable Zero-Shot Prediction of the Effects of Mutations on Protein Function. Adv. Neural Inf. Process. Syst..

[54] Hie B.L., Shanker V.R., Xu D., Bruun T.U., Weidenbacher P.A., Tang S., Wu W., Pak J.E., Kim P.S. (2024). Efficient Evolution of Human Antibodies from General Protein Language Models. Nat. Biotechnol..

[55] Shanker V.R., Bruun T.U.J., Hie B.L., Kim P.S. (2024). Unsupervised Evolution of Protein and Antibody Complexes with a Structure-Informed Language Model. Science.

[56] Gao S., Yang W., Zheng X., Wang T., Zhang D., Zou X. (2025). Advances of Nanobody-Based Immunosensors for Detecting Food Contaminants. Trends Food Sci. Technol..

[57] Ahmad M.I., Amorim C.G., Qatouseh L.F.A., Montenegro M.C. (2024). Nanobody-Based Immunodiagnostics: A Systematic Review of Nanobody Integration in Diagnostics and Deep Insight into Electrochemical Immunoassays. Microchem. J..

[58] Ramon A., Ali M., Atkinson M., Saturnino A., Didi K., Visentin C., Ricagno S., Xu X., Greenig M., Sormanni P. (2024). Assessing Antibody and Nanobody Nativeness for Hit Selection and Humanization with AbNatiV. Nat. Mach. Intell..

[59] Deng J., Gu M., Zhang P., Dong M., Liu T., Zhang Y., Liu M. (2024). Nanobody–Antigen Interaction Prediction with Ensemble Deep Learning and Prompt-Based Protein Language Models. Nat. Mach. Intell..

[60] Douaki A., Garoli D., Inam A.K.M.S., Angeli M.A.C., Cantarella G., Rocchia W., Wang J., Petti L., Lugli P. (2022). Smart Approach for the Design of Highly Selective Aptamer-Based Biosensors. Biosensors.

[61] Yu T., Cui H., Li J.C., Luo Y., Jiang G., Zhao H. (2023). Enzyme Function Prediction Using Contrastive Learning. Science.

[62] Tuta-Navajas G.H., Roa-Prada S., Chalela-Alvarez G. (2022). Kinetic Model Parameter Estimation Using Genetic Algorithms of the Oxidation of Phenol in Water Catalyzed by the Laccase Enzyme for the Design of a Biosensor. Bioremediat. J..

[63] Bachas S., Rakocevic G., Spencer D., Sastry A.V., Haile R., Sutton J.M., Kasun G., Stachyra A., Gutierrez J.M., Yassine E. (2022). Antibody Optimization Enabled by Artificial Intelligence Predictions of Binding Affinity and Naturalness. bioRxiv.

[64] Li B.-Q., Zhang Y.-C., Huang G.-H., Cui W.-R., Zhang N., Cai Y.-D. (2014). Prediction of Aptamer-Target Interacting Pairs with Pseudo-Amino Acid Composition. PLoS ONE.

[65] Yu H., Deng H., He J., Keasling J.D., Luo X. (2023). UniKP: A Unified Framework for the Prediction of Enzyme Kinetic Parameters. Nat. Commun..

[66] LeCuyer K.A., Crothers D.M. (1994). Kinetics of an RNA Conformational Switch. Proc. Natl. Acad. Sci. USA.

[67] Umuhire Juru A., Patwardhan N.N., Hargrove A.E. (2019). Understanding the Contributions of Conformational Changes, Thermodynamics, and Kinetics of RNA–Small Molecule Interactions. ACS Chem. Biol..

[68] Wang F., Li W., Li B., Xie L., Tong Y., Xu X. (2023). cRNAsp12 Web Server for the Prediction of Circular RNA Secondary Structures and Stabilities. Int. J. Mol. Sci..

[69] Soylu N.N., Sefer E. (2023). Bert2ome: Prediction of 2′-O-Methylation Modifications from Rna Sequence by Transformer Architecture Based on Bert. IEEE/ACM Trans. Comput. Biol. Bioinform..

[70] Yang M., Xie C., Lu H. (2025). Advances in MXene-Based Electrochemical Sensors for Multiplexed Detection in Biofluids. Int. J. Mol. Sci..

[71] Li Z., Guo M., Zhong W. (2025). Multiplex Detection of Biomarkers Empowered by Nanomaterials. Precis. Chem..

[72] Chen Y., Zhang X., Liu Y., Liu Z., Li Z., Li Z., Chen X., Liu J., Chen Z., Mo G. (2025). A Gold Nanoparticles and MXene Nanocomposite Based Electrochemical Sensor for Point-of-Care Monitoring of Serum Biomarkers. ACS Nano.

[73] Ranjan P., Abubakar Sadique M., Yadav S., Khan R. (2022). An Electrochemical Immunosensor Based on Gold-Graphene Oxide Nanocomposites with Ionic Liquid for Detecting the Breast Cancer CD44 Biomarker. ACS Appl. Mater. Interfaces.

[74] Lu X., Liu P., Bisetty K., Cai Y., Duan X., Wen Y., Zhu Y., Rao L., Xu Q., Xu J. (2022). An Emerging Machine Learning Strategy for Electrochemical Sensor and Supercapacitor Using Carbonized Metal–Organic Framework. J. Electroanal. Chem..

[75] Kavya K.V., Kumar R.S., Rajendra Kumar R.T., Ramesh S., Yang W., Kakani V., Haldorai Y. (2024). Fabrication of 1D/2D Au Nanofiber/MIL-101(Cr)–NH2 Composite for Selective Electrochemical Detection of Caffeic Acid: Predicting Sensor Performance by Machine Learning and Investigating the Porosity Using AI and Computer Vision-Based Image Analysis. Microchem. J..

[76] Nezhadali A., Bonakdar G.A. (2019). Multivariate Optimization of Mebeverine Analysis Using Molecularly Imprinted Polymer Electrochemical Sensor Based on Silver Nanoparticles. J. Food Drug Anal..

[77] Imani A., Hosseinpour S., Keyhani A., Azimzadeh M. (2020). Modeling and Optimization of Oligonucleotide-Based Nanobiosensor Using Artificial Neural Network and Genetic Algorithm Based Procedure. Iran. J. Biosyst. Eng..

[78] Yarahmadi B., Hashemianzadeh S.M., Milani Hosseini S.M.-R. (2023). Machine-Learning-Based Predictions of Imprinting Quality Using Ensemble and Non-Linear Regression Algorithms. Sci. Rep..

[79] Li D., Zhou H., Hui X., He X., Mu X. (2021). Plasmonic Biosensor Augmented by a Genetic Algorithm for Ultra-Rapid, Label-Free, and Multi-Functional Detection of COVID-19. Anal. Chem..

[80] Qiao D., Zhai T., Liu J.-M., Wang S. (2025). Machine Learning-Based Smart Technology Enables Precise and Efficient Detection of Food Safety Risks. Food Rev. Int..

[81] Zhong Y.H., Zhang S., He R., Zhang J., Zhou Z., Cheng X., Huang G., Zhang J. (2019). A Convolutional Neural Network Based Auto Features Extraction Method for Tea Classification with Electronic Tongue. Appl. Sci..

[82] Gundry L., Guo S.-X., Kennedy G., Keith J., Robinson M., Gavaghan D., Bond A.M., Zhang J. (2021). Recent Advances and Future Perspectives for Automated Parameterisation, Bayesian Inference and Machine Learning in Voltammetry. Chem. Commun..

[83] Zhu S., Jiang K., Chen B., Zheng S. (2023). Data-Driven Design of Electrocatalysts: Principle, Progress, and Perspective. J. Mater. Chem. A.

[84] Xu Y., Li C., Jiang Y., Guo M., Yang Y., Yang Y., Yu H. (2020). Electrochemical Impedance Spectroscopic Detection of *E. coli* with Machine Learning. J. Electrochem. Soc..

[85] Ccopa Rivera E., Swerdlow J.J., Summerscales R.L., Uppala P.P.T., Maciel Filho R., Neto M.R.C., Kwon H.J. (2020). Data-Driven Modeling of Smartphone-Based Electrochemiluminescence Sensor Data Using Artificial Intelligence. Sensors.

[86] Hanssen B.L., Siraj S., Wong D.K.Y. (2016). Recent Strategies to Minimise Fouling in Electrochemical Detection Systems. Rev. Anal. Chem..

[87] Aiassa S., Ny Hanitra I., Sandri G., Totu T., Grassi F., Criscuolo F., De Micheli G., Carrara S., Demarchi D. (2021). Continuous Monitoring of Propofol in Human Serum with Fouling Compensation by Support Vector Classifier. Biosens. Bioelectron..

[88] Curulli A. (2020). Nanomaterials in Electrochemical Sensing Area: Applications and Challenges in Food Analysis. Molecules.

[89] Jawaid S., Sharma B.P., Hussain Tumrani S., Abbas Z., Ali Soomro R., Karakuş S., Küçükdeniz T., Nafady A. (2024). Enhanced Electrochemical Oxidation and Machine Learning-Assisted Sensing of Tetrabromobisphenol A Using Activated Carbon Facilitated CoWO4 Heterostructures. Mater. Sci. Eng. B.

[90] Gu B.-C., Chung K.-J., Chen B.-W., Dai Y.-H., Wu C.-C. (2023). Electrochemical Detection Combined with Artificial Neural Networks for the Simultaneous Intelligent Sensing of Caffeine and Chlorogenic Acid. Electrochim. Acta.

[91] Bonet-San-Emeterio M., González-Calabuig A., del Valle M. (2019). Artificial Neural Networks for the Resolution of Dopamine and Serotonin Complex Mixtures Using a Graphene-Modified Carbon Electrode. Electroanalysis.

[92] Torrecilla J.S., Mena M.L., Yáñez-Sedeño P., García J. (2008). A Neural Network Approach Based on Gold-Nanoparticle Enzyme Biosensor. J. Chemom..

[93] Lee K., Ha S.M., Gurudatt N.G., Heo W., Hyun K.-A., Kim J., Jung H.-I. (2024). Machine Learning-Powered Electrochemical Aptasensor for Simultaneous Monitoring of Di(2-Ethylhexyl) Phthalate and Bisphenol A in Variable pH Environments. J. Hazard. Mater..

[94] Madakam S., Ramaswamy R., Tripathi S. (2015). Internet of Things (IoT): A Literature Review. J. Comput. Commun..

[95] Bocu R. (2024). Extended Review Concerning the Integration of Electrochemical Biosensors into Modern IoT and Wearable Devices. Biosensors.

[96] Çıtmacı B., Luo J., Jang J.B., Korambath P., Morales-Guio C.G., Davis J.F., Christofides P.D. (2022). Digitalization of an Experimental Electrochemical Reactor via the Smart Manufacturing Innovation Platform. Digit. Chem. Eng..

[97] Diamond D., Coyle S., Scarmagnani S., Hayes J. (2008). Wireless Sensor Networks and Chemo-/Biosensing. Chem. Rev..

[98] Piyare R., Lee S.R. (2013). Towards Internet of Things (IOTS): Integration of Wireless Sensor Network to Cloud Services for Data Collection and Sharing. Int. J. Comput. Netw. Commun..

[99] Khalaf E.M., Sanaan Jabbar H., Mireya Romero-Parra R., Raheem Lateef Al-Awsi G., Setia Budi H., Altamimi A.S., Abdulfadhil Gatea M., Falih K.T., Singh K., Alkhuzai K.A. (2023). Smartphone-Assisted Microfluidic Sensor as an Intelligent Device for on-Site Determination of Food Contaminants: Developments and Applications. Microchem. J..

[100] Bianchi V., Boni A., Bassoli M., Giannetto M., Fortunati S., Careri M., De Munari I. (2021). IoT and Biosensors: A Smart Portable Potentiostat with Advanced Cloud-Enabled Features. IEEE Access.

[101] Kaushik A.K., Dhau J.S., Gohel H., Mishra Y.K., Kateb B., Kim N.-Y., Goswami D.Y. (2020). Electrochemical SARS-CoV-2 Sensing at Point-of-Care and Artificial Intelligence for Intelligent COVID-19 Management. ACS Appl. Bio Mater..

[102] Beduk D., Ilton de Oliveira Filho J., Beduk T., Harmanci D., Zihnioglu F., Cicek C., Sertoz R., Arda B., Goksel T., Turhan K. (2022). “All In One” SARS-CoV-2 Variant Recognition Platform: Machine Learning-Enabled Point of Care Diagnostics. Biosens. Bioelectron. X.

[103] Liang Y., Lee M.H., Zhou A., Khanthaphixay B., Hwang D.S., Yoon J.-Y. (2023). eXtreme Gradient Boosting-Based Classification of Bacterial Mixtures in Water and Milk Using Wireless Microscopic Imaging of Quorum Sensing Peptide-Conjugated Particles. Biosens. Bioelectron..

